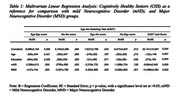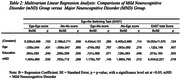# The Ego‐Allo Switching Task for Assessing Visuospatial Memory in Brazilian Old Adults

**DOI:** 10.1002/alz.089025

**Published:** 2025-01-03

**Authors:** Victoria Sciascia Cetraro, Ana Beatriz Silva, Ana Luiza Nunes Cunha, Carolina Pinto Souza, Vitor Tumas, Maria Paula Foss

**Affiliations:** ^1^ Faculty of Philosophy, Sciences, and Letters of the University of São Paulo at Ribeirão Preto (FFCLRP‐USP), Ribeirão Preto, São Paulo Brazil; ^2^ Clinical Hospital of the Ribeirão Preto Medical School of the University of São Paulo (HCFMRP‐USP), Ribeirão Preto, São Paulo Brazil

## Abstract

**Background:**

Spatial orientation involves egocentric and allocentric strategies that switch in the brain. Disturbances in switching may indicate Neurocognitive Disorders, which contribute to early detection of Alzheimer’s Disease. The “Ego‐Allo‐Switching Task” (EAST) needs to be adapted for cross‐cultural use in Brazil.

**Method:**

61 participants were divided into Cognitively Healthy Seniors (CHS; n = 21), mild Neurocognitive Disorder (mND; n = 20), and Major Neurocognitive Disorder (MND; n = 20). The EAST was applied to all participants, and CHS individuals were retested within 1‐2 weeks. In this study, we modified the original EAST to better suit Brazilian older adults, considering their unfamiliarity with specific geometric shapes. Thus, we developed an adapted version focused on shape recognition rather than free recall. Before the test, two examiners evaluated all participants using the Clinical Dementia Rating (CDR) to ensure they fit into one of the three groups. The test‐retest reliability was analyzed by intraclass correlation coefficient. Additionally, demographics (age and education) were compared between groups using the Kruskal‐Wallis test, followed by Dunn’s post hoc test and a multivariate linear regression analysis was conducted. All data were analyzed with SPSS 17.0.

**Result:**

The global results of the EAST demonstrated high consistency in the test‐retest comparison (ICCs = 0.84). Initial discrepancies in age and education among CHS, mND, and MND groups (p < 0.001) were corrected using multivariate linear regression. The analysis highlighted distinct EAST performances between groups, as follows: ego‐ego (CHS > mND > MND); alo‐alo (CHS > (mND and MND)); ego‐alo (CHS > (mND e MND)); alo‐ego ((CHS e mND) > MND); EAST total score (CHS > mMD > MND) (Table 1 and 2).

**Conclusion:**

EAST detects changes in spatial orientation skills related to neurodegenerative diseases over time and has temporal reliability. Future research should expand its psychometric properties to Brazilian seniors. This study fills the gap in neuropsychological measures for detecting task‐switching ability.